# Ultradispersed Cobalt Ferrite Nanoparticles Assembled in Graphene Aerogel for Continuous Photo-Fenton Reaction and Enhanced Lithium Storage Performance

**DOI:** 10.1038/srep29099

**Published:** 2016-07-04

**Authors:** Bocheng Qiu, Yuanxin Deng, Mengmeng Du, Mingyang Xing, Jinlong Zhang

**Affiliations:** 1 Key Laboratory for Advanced Materials and Institute of Fine Chemicals, East China University of Science and Technology, 130 Meilong Road, Shanghai 200237, P.R. China

## Abstract

The Photo-Fenton reaction is an advanced technology to eliminate organic pollutants in environmental chemistry. Moreover, the conversion rate of Fe^3+^/Fe^2+^ and utilization rate of H_2_O_2_ are significant factors in Photo-Fenton reaction. In this work, we reported three dimensional (3D) hierarchical cobalt ferrite/graphene aerogels (CoFe_2_O_4_/GAs) composites by the *in situ* growing CoFe_2_O_4_ crystal seeds on the graphene oxide (GO) followed by the hydrothermal process. The resulting CoFe_2_O_4_/GAs composites demonstrated 3D hierarchical pore structure with mesopores (14~18 nm), macropores (50~125 nm), and a remarkable surface area (177.8 m^2 ^g^−1^). These properties endowed this hybrid with the high and recyclable Photo-Fenton activity for methyl orange pollutant degradation. More importantly, the CoFe_2_O_4_/GAs composites can keep high Photo-Fenton activity in a wide pH. Besides, the CoFe_2_O_4_/GAs composites also exhibited excellent cyclic performance and good rate capability. The 3D framework can not only effectively prevent the volume expansion and aggregation of CoFe_2_O_4_ nanoparticles during the charge/discharge processes for Lithium-ion batteries (LIBs), but also shorten lithium ions and electron diffusion length in 3D pathways. These results indicated a broaden application prospect of 3D-graphene based hybrids in wastewater treatment and energy storage.

Three dimensional (3D) graphene aerogels (GAs) with hierarchical porous structure have been attracting increasing attention in different fields, such as sensors^1^,^2^,^3^, oil absorption^4^,^5^,^6^, energy storage^7^,^8^,^9^, and catalysis^10^,^11^. These porous GAs not only inherit the intriguing properties of two-dimensional (2D) graphene sheet including excellent electrical conductivity and high surface area^12^,^13^,^14^,^15^,^16^,^17^, but also endow graphene with controllable macro-appearance, high elastic property, adjustable porosity and ultralow density. More than these properties, the GAs building block can promote the separation of photogenerated electrons and holes, which can drastically enhance the performance of photocatalysts^18^. All these properties of GAs make it especially appealing as an ideal support to load various active components such as metal^19^,^20^, metal sulfides^21^, and metal oxides^22^,^23^,^24^. Recently, considerable efforts have been to devote to the development of 3D graphene-based composites for Lithium-ion batteries (LIBs) and catalysis^25^,^26^,^27^,^28^. Huang *et al*. have pioneered the capture of SnO_2_ into the 3D graphene frameworks by amphiphilic polymer-promoted assembly method and the resulting SnO_2_/graphene frameworks with controllable macroporous structures show the unprecedented high capacity and excellent cycle performance in LIBs^29^. Our research group has reported a simple one-step hydrothermal method for the preparation of ultradispersed TiO_2_ single nanocrystals grown *in situ* on the aerogel surface and the as-prepared TiO_2_/GAs composites have highly recyclable photocatalytic activity, a high rate capability, and stable cycling in LIBs^18^. In order to extend the application of GAs in the environmental issues, the Fenton-reagent of Fe_2_O_3_/GAs composites were successfully prepared by a Stöber-like method, which displayed an ultrastable solar-driven Fenton activity over a wide pH range of 3.5–9.0^30^. Different from above mentioned simple oxides, the composites based the mixed oxides and aerogels have been rarely reported. CoFe_2_O_4_ is a typical mixed oxide with potential Fenton-induced activity and Li^+^ storage property^31^,^32^,^33^. On the other hand, CoFe_2_O_4_ as a kind of magnetic materials has been extensively studied due to its excellent chemical and mechanical stability^34^, high coercive force^35^, and potential applications in the fields of environment treatment^36^, bioseparation and magnetic resonance imaging^37^,^38^.

In this work, we employed a combined hydrothermal self-assembly and freeze-drying technology to construct the CoFe_2_O_4_/GAs composites with mesoporous and macroporous structure. Without any surfactant, ultradispersed CoFe_2_O_4_ nanoparticles and supporting 3D graphene network are simultaneously synthesized through a hydrothermal process using CoFe_2_O_4_ crystal seeds loaded on the surface of graphene oxide (GO) sheets as the basic building block. Compared with the mechanically mixed CoFe_2_O_4_/reduced graphene oxide (CoFe_2_O_4_/RGO) composites, the CoFe_2_O_4_/GAs composites demonstrate the 3D interconnected porous structure with a uniform deposition of CoFe_2_O_4_ nanoparticles, which can effectively capture electron to facilitate the Fe^3+^/Fe^2+^ conversion in Photo-Fenton reaction. Thereby, the CoFe_2_O_4_/GAs composites show a high Photo-Fenton activity for degradation of methyl orange pollutant. Besides, the 3D porous structure provides the short diffusion length, excellent conductive network and high surface area for lithium ions transport. As a result, the CoFe_2_O_4_/GAs composites exhibit excellent cyclic performance (830 mA h g^−1^ for up to 50 charge/discharge cycles at a current density of 0.1 A g^−1^) and good rate capability (830 and 340 mA h g^−1^ at 0.1 and 2.0 A g^−1^, respectively).

## Results

**Figure 1:**
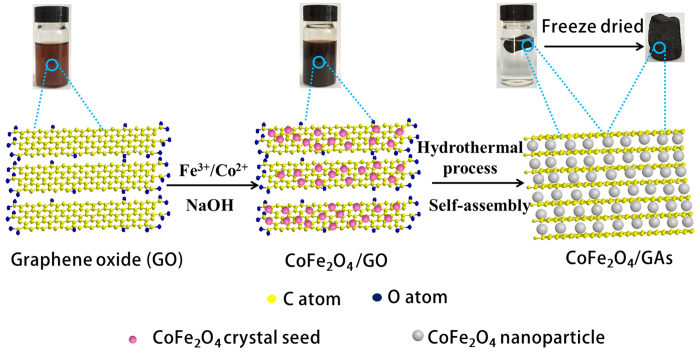
Fabrication process for CoFe_2_O_4_/GAs.

The overall fabrication procedure of CoFe_2_O_4_/GAs is illustrated in Fig. 1. Firstly, iron nitrate hydrate (Fe(NO_3_)_3_•9H_2_O) and cobalt nitrate hydrate (Co(NO_3_)_2_•6H_2_O) are dissolved in the graphene oxide (GO) suspension at room temperature. During the process, positively charged Fe^3+^ and Co^2+^ can be absorbed to the hydroxyl and carboxyl groups on the surface of the negatively charged GO sheet by electrostatic attraction. The controllable nucleation site of CoFe_2_O_4_ on the GO sheet can be realized by the addition of sodium hydroxide (NaOH) solution. That is, upon the addition of NaOH solution, the hydrolysis of Fe^3+^ and Co^2+^ leads to the formation of CoFe_2_O_4_ crystal seeds deposited on the surface of GO sheets. This result can be confirmed by the HRTEM images of CoFe_2_O_4_/GO. As shown in Figure S1a,b, a large number of CoFe_2_O_4_ crystal seeds with a size of ~3 nm are highly dispersed on the GO sheets. Thereafter, the 2D GO sheets with a uniform decoration of CoFe_2_O_4_ crystal seeds self-assemble into the 3D monolithic networks during hydrothermal treatment, where reduction of GO sheets and crystallization and growth of CoFe_2_O_4_ crystal seeds are simultaneously realized. Finally, the CoFe_2_O_4_/GAs composites are obtained through the lyophilization. As a control experiment, the two-dimensional (2D) CoFe_2_O_4_/reduced graphene oxide (RGO) composites are prepared by physically mixing CoFe_2_O_4_ and RGO, denoted as CoFe_2_O_4_/RGO.

**Figure 2:**
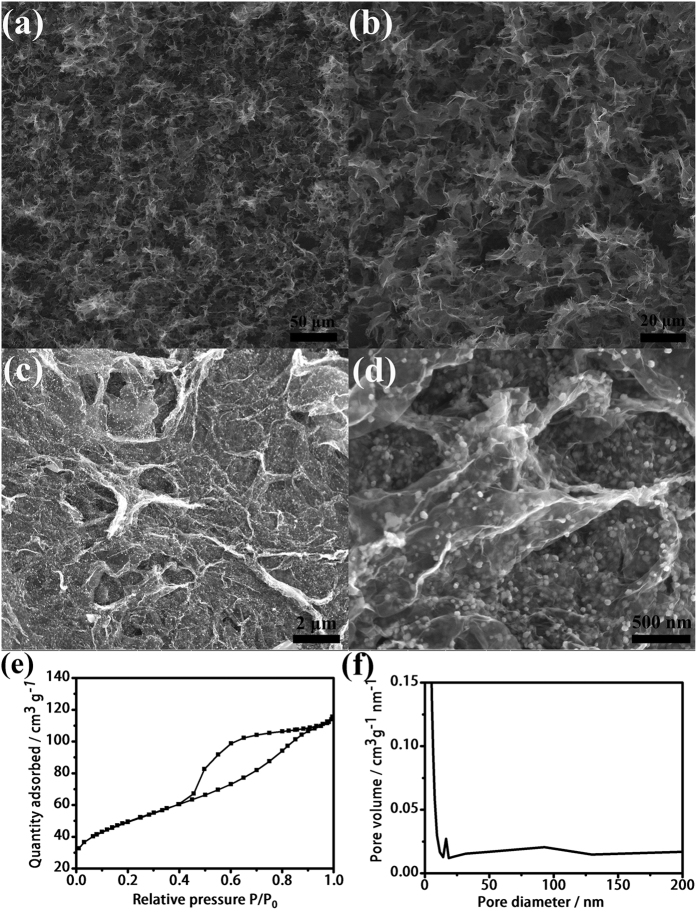
SEM and FESEM images. (**a**,**b**) SEM and (**c**,**d**) FESEM images of CoFe_2_O_4_/GAs. Nitrogen adsorption/desorption isotherms (**e**) and pore size distribution (**f**) of CoFe_2_O_4_/GAs.

The morphology and microstructure of the resulting CoFe_2_O_4_/GAs composites were elucidated by scanning electron microscopy (SEM), field emission scanning electron microscopy (FESEM) and nitrogen adsorption/desorption analysis. As shown in Fig. 2a,b, the CoFe_2_O_4_/GAs composites show macroporous structure with well-defined interconnected pores at micrometer order. The partial overlapping or coalescence of the graphene sheet led to the physically cross-linked sites in the CoFe_2_O_4_/GAs composites. The driving force for assembly of 3D porous interconnected framework in CoFe_2_O_4_/GAs through the hydrothermal process should be ascribed to π-π interaction between graphene sheets. The FESEM images of CoFe_2_O_4_/GAs (Fig. 2c,d) exhibit that all the CoFe_2_O_4_ nanoparticles with a size of around 9 nm are highly dispersed on the surface of RGO sheets. It is noteworthy that some CoFe_2_O_4_ nanoparticles can be encapsulated within the RGO sheets (Fig. 2d), which can effectively prevent the layer-by-layer stacking of GO sheets during the reduction process and avoid direct connect between CoFe_2_O_4_ and electrolyte. The mesoporous nature of the CoFe_2_O_4_/GAs composites was confirmed by nitrogen adsorption/desorption analysis. The adsorption data reveal a remarkably high specific surface area of 177.8 m^2 ^g^−1^ (Fig. 2e), and the pore size distribution curve indicates the presence of hierarchical porous structure (Fig. 2f). The mesoporous size is in the range of 14~18 nm, and the macroporous size is in a wide range of 50~125 nm. This result highlights that the building up of 3D-GAs by hydrothermal method is an effective way to achieve a high surface area and hierarchical porous structure for 3D graphene-based materials.

**Figure 3:**
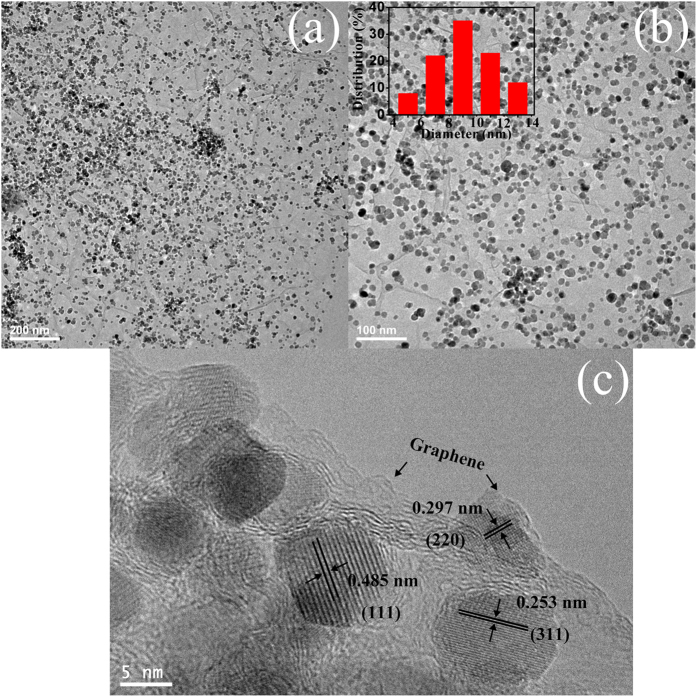
TEM and HRTEM images. (**a**,**b**) TEM images and (**c**) HRTEM image of CoFe_2_O_4_/GAs. Inset b is the corresponding particle size distribution of the loaded CoFe_2_O_4_ nanoparticles derived from 100 of CoFe_2_O_4_ particles in image (**b**).

TEM and HRTEM characterizations were conducted to obtain a closer morphology and structure of the CoFe_2_O_4_/GAs composites. The low-resolution TEM image (Fig. 3a) of the CoFe_2_O_4_/GAs composites exhibits that CoFe_2_O_4_ nanoparticles are uniformly deposited on the ultrathin RGO sheets, which is in good agreement with the FESEM result. Importantly, no obvious large and aggregated CoFe_2_O_4_ nanoparticles are visible, and no naked GO sheets or free CoFe_2_O_4_ nanoparticles appear. In addition, the TEM image (Fig. 3b) further reveals that a large number of CoFe_2_O_4_ nanoparticles are highly dispersed on the surface of RGO sheets. The size distribution curve of CoFe_2_O_4_ nanoparticles shows an average size focused on around 9 nm (Fig. 3b, inset). The HRTEM image (Fig. 3c) demonstrates that the highly crystalline CoFe_2_O_4_ nanoparticles are randomly distributed on two sides of RGO sheets with different contrasts. Moreover, the edge of RGO sheets can be clearly observed as indicated by the arrow (Fig. 3c) and some individual CoFe_2_O_4_ nanoparticles display clear crystal lattice with three kinds of spacing of 0.253 nm, 0.485 nm and 0.297 nm corresponding to the (311), (111) and (220) plane, respectively^31^. Elemental mapping analysis of the CoFe_2_O_4_/GAs composites is performed to illustrate the distribution of carbon, cobalt, iron, and oxygen components in the composites (Figure S2). Apparently, the carbon, cobalt, iron, and oxygen components are uniformly distributed on RGO sheets, further verifying the ultradispersed distribution of CoFe_2_O_4_ nanoparticles on the surface of RGO sheets.

**Figure 4:**
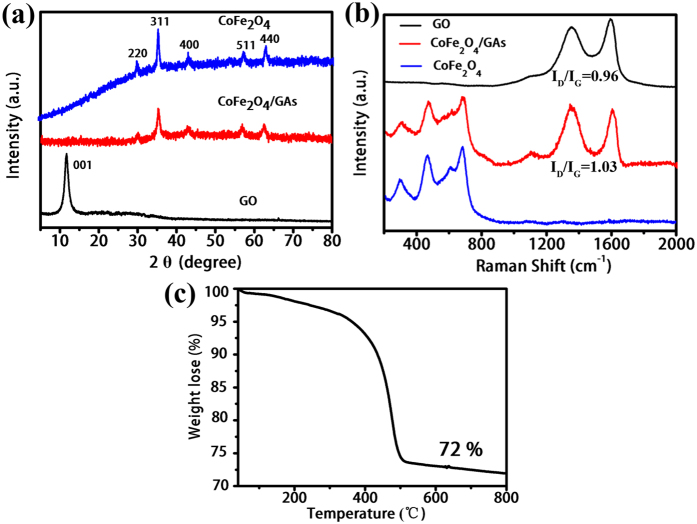
XRD patterns and TGA analysis. (**a**) XRD patterns of CoFe_2_O_4_, CoFe_2_O_4_/GAs and GO. (**b**) Raman spectra of GO, CoFe_2_O_4_/GAs and CoFe_2_O_4_. (**c**) Thermogravimetric analysis (TGA) curves of CoFe_2_O_4_/GAs composites in air from 40–800 °C with a heating rate of 20 °C min^−1^.

The XRD patterns of the as-prepared CoFe_2_O_4_/GAs depicted in Fig. 4a show diffraction peaks at 2θ = 30.1°, 35.4°, 43.1°, 57.1°, 62.7°, which correspond to the crystal indexes of (220), (311), (400), (511), and (440) plane, respectively. All the diffraction peaks are completely consistent with the peaks of commercial CoFe_2_O_4_, indicating that the CoFe_2_O_4_ nanoparticles grown on the RGO sheets are well crystallized after the hydrothermal treatment. The presence of characteristic peaks in Raman spectra (Fig. 4b) also confirm the generation of highly crystallized CoFe_2_O_4_ on the RGO sheets. Moreover, the diffraction (001) reflection at 2θ = 11.7° of the initial GO sheet can be observed, but no corresponding diffraction peak can be observed in the XRD patterns of CoFe_2_O_4_/GAs, indicating the reduction of GO under the hydrothermal treatment. These results suggest the reduction of GO sheets and the crystallization of CoFe_2_O_4_ nanoparticles are proceed simultaneously. In addition, the obvious increasement of the intensity ratio of D/G bands through the hydrothermal process in the Raman spectra further confirms the reduction of GO (D/G ratio increases from 0.96 to 1.03, Fig. 4b). TGA measurement carried out in the air was used to determine the mass fraction of CoFe_2_O_4_ in the composites. As shown in Fig. 4c, the TGA curve displays a significant loss weight at approximately 450 °C. The miniscule weight loss (<3%) that appeared below 300 °C is most likely attributed to the evaporation of water molecules adsorbed into the 3D interconnected networks. The major weight loss from 300 to 500 °C was about 20%, indicating the combustion of RGO. Therefore, the CoFe_2_O_4_/GAs composites contained about 72% (w/w) of CoFe_2_O_4_.

## Discussion

The Fenton processes for waste water treatment have attracted more attention because of the formation of hydroxyl radicals (•OH) during degradation^39^. Actually, the generated •OH radicals are highly active and nonselective, and they are able to decompose many non-biolodegradable and persistent organic compounds^40^. Iron-containing materials^41^, other transitional metals^42^, or nonmetallic materials exhibit catalytic activity for the Fenton reaction. In addition, electro-, sono-, photo-assisted Fenton reaction, or to say, an integration technology, have been widely studied as well^43^.

**Figure 5:**
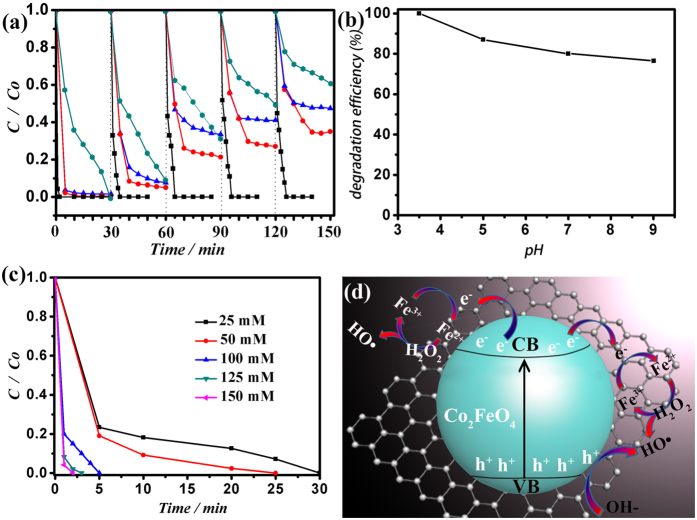
Photo-Fenton tests. Cycle test for the solar-driven degradation of methyl-orange (black line: CoFe_2_O_4_/GAs with H_2_O_2_ under irradiation; red line: CoFe_2_O_4_/GAs with H_2_O_2_ in the dark; blue line: CoFe_2_O_4_/GAs without H_2_O_2_ in the dark; dark cyan line: pure CoFe_2_O_4_ powders with H_2_O_2_ under irradiation) (70 mL MO, 10 mg/L) under simulated solar light irradiation (with an AM 1.5 air mass filter) (150 mM H_2_O_2_ (30 wt%), the initial pH was 3.5) (**a**). Effect of solution pH on photodegradation efficiency of MO on CoFe_2_O_4_/GAs photocatalyst (70 mL MO, 10 mg/L; 150 mM H_2_O_2_ (30 wt%); t:30 min; the third cycle data) (**b**). Effect of H_2_O_2_ concentration on photodegradation efficiency of MO on CoFe_2_O_4_/GAs photocatalyst (70 mL MO, 10 mg/L; pH: 3.5; the third cycle data) (**c**). Photo-Fenton reaction mechanism of CoFe_2_O_4_/GAs (**d**).

In this study, Photo-Fenton reactions are conducted for methyl orange (MO 10 mg/L) degradation to test the activity of CoFe_2_O_4_/GAs. The hydrochloric acid (HCl 0.1 M) is used to adjust the pH value of the reaction system. The reaction is proceeded under the illumination of a 300 W Xenon lamp by an AM 1.5 G solar simulator. It is noteworthy, on the other hand, to highlight the fact that the CoFe_2_O_4_/GAs composites were grinded to powders in order to increase their contact area with the H_2_O_2_ molecules during the Photo-Fenton reaction, thereby improving the utilization efficiency of H_2_O_2_. As shown in Fig. 5a, the CoFe_2_O_4_/GAs composites in the dark show superior adsorption capacity in the first cycle test and all the MO molecules are absorbed in 1 min. Thereafter, the adsorption capacity gradually decreased after 5 cycles, but 65% of the MO molecules can still be adsorbed in 30 min, which reveals the good adsorption capacity of CoFe_2_O_4_/GAs. With the addition of H_2_O_2_ in the dark, the decrement of MO content is caused by the adsorption and Fenton-like reaction. However, the Fenton-like reaction activity still decreased after 5 cycles, which suggests that the conversion efficiency of Fe^3+^/Fe^2+^ in the Fenton-like reaction without the aid of light is very low. So we introduce light into the Fenton-like reaction. As shown in Fig. 5a, the activity with photo-assisted has been improved greatly. Importantly, the activity keeps almost unchanged after 5 cycles, indicating the high conversion efficiency of Fe^3+^/Fe^2+^. For comparsion, pure CoFe_2_O_4_ nanoparticles are prepared and keep a good dispersed state (Figure S3). Seen from Fig. 4a, pure CoFe_2_O_4_ shows decreased Photo-Fenton activity after 5 cycles due to low conversion efficiency of Fe^3+^/Fe^2+^ and leaching of Fe^2+^. Furthermore, we used 1, 10-phenanthroline monohydrate (Phen) as a testing Fe^2+^ reagent to detect the leaching of Fe^2+^ (Figure S4). The Fe^2+^ ions can react with the Phen to generate a strong visible absorption signal. After adding with Phen, the reaction solution of the CoFe_2_O_4_ powders gives a strong visible absorption signal, but the reaction solution of CoFe_2_O_4_/GAs gives a very low visible absorption signal, which indicates the leaching of Fe^2+^ ions in the aqueous solution is low. To further highlight the structure stability of CoFe_2_O_4_/GAs, we observe the morphology of the catalyst after 5 cycles. As shown in Figure S5, all the CoFe_2_O_4_ particles are still ultra-dispersed on the surface of RGO sheets (Figure S5a,b) and the 3D porous structure can be observed clearly (Figure S5c,d), which further reveals the high stability of structures. Figure S6 shows ferromagnetic property of the as-prepared CoFe_2_O_4_/GAs composites, suggesting that such composites might be easily separated from solution phase through inducing an external magnetic field.

The pH of the solution plays a key role in Photo-Fenton degradation of pollutants^44^. The MO solution can be degraded with CoFe_2_O_4_/GAs within pH 3.5–9 (Fig. 5b). In order to excluding the strong adsorption of MO (Fig. 5a), we conducted cycle tests and selected the data of the third cycle test of CoFe_2_O_4_/GAs under different pH. It can be observed that the degradation rate decreases a little when pH is increased from 3.5 to 9, which is in good agreement with the previous reports^30^,^45^. When pH is adjusted to 9, the Photo-Fenton degradation rate is up to 78% in 30 min. In addition, the H_2_O_2_ concentration on the rate of degradation of MO was also investigated by varying the H_2_O_2_ concentration from 25 to 150 mM (Fig. 5c). We also conducted cycle tests and selected the data of the third cycle test of CoFe_2_O_4_/GAs under different H_2_O_2_ concentration. Figure 5c shows the variation in the rate constants with H_2_O_2_ concentration in the presence of the catalyst. It can be seen that with the increasing of H_2_O_2_ concentration, the degradation rate of MO can be correspondingly improved. The enhanced Photo-Fenton activity is expected due to the increasement of HO▪ yield from H_2_O_2_ reacted with Fe^3+^. Under a relative low H_2_O_2_ concentration (25 mM), all the MO molecules can be degraded in 30 min, exhibiting the high Photo-Fenton activity. Figure 5d demonstrates schematic representation of the Photo-Fenton reaction in the CoFe_2_O_4_/GAs composites. Firstly, the electron-hole pairs from CoFe_2_O_4_ are generated under simulated solar light irradiation (Eq. (1)). The photogenerated electrons are quickly trapped by graphene (Eq. (2)), limiting the recombination of holes and electrons. At the same time, the photogenerated holes (h^+^) are subsequently trapped by OH- to produce ▪OH radicals. The electrons trapped by graphene can be used to reduce Fe^3+^ to form Fe^2+^ (Eq. (3)). The Fe^2+^ can react with H_2_O_2_ to form 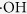 radical and Fe^3+^ (Eq. (4))^30^. The generated Fe^3+^ can be reduced to Fe^2+^ again by the electron concentrated on the surface of RGO sheets to keep the cycle of Fe^3+^/Fe^2+^, thus achieving the high Photo-Fenton activity.


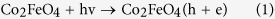



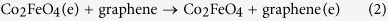



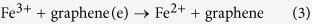



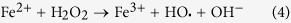


**Figure 6:**
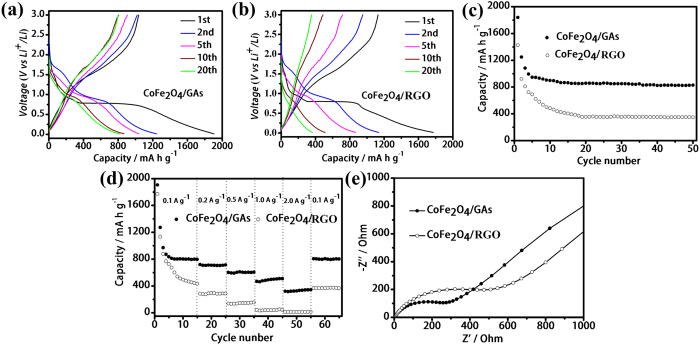
LIBs tests. The charge/discharge curves of CoFe_2_O_4_/GAs (**a**) and mechanically mixed CoFe_2_O_4_/GR (**b**) electrodes at constant current densities of 0.1 A g^−1^. Cycling performance of CoFe_2_O_4_/GAs composites and CoFe_2_O_4_/GR composites electrode at constant current densities of 0.1 A g^−1^ (**c**). Rate capability of CoFe_2_O_4_/GAs composites and CoFe_2_O_4_/RGO composites at each current density between 0.1 and 2 A g^−1^ (**d**). Nyquist plots of the electrodes of CoFe_2_O_4_/GAs and CoFe_2_O_4_/RGO composites. All of the measurements were conducted using a voltage window of 0.01–3.0 V (**e**).

On the other hand, the lithium-insertion/extraction properties of the CoFe_2_O_4_/GAs composites as anode material were investigated by galvanostatic charge/discharge measurements over a voltage range of 0.01–3.0 V. Figure 6a shows the charge/discharge curve of CoFe_2_O_4_/GAs at a current density of 0.1 A g^−1^. In the first discharge step, the CoFe_2_O_4_/GAs composites present an extended/long voltage plateau at about 0.8 V, followed by a sloping curve down to the cut off voltage of 0.01 V, which is a typical characteristic of voltage trend for the CoFe_2_O_4_ electrode^31^,^46^. A high initial reversible capacity of 1905 mA h g^−1^ can be derived in the first discharge step, with a corresponding charge capacity of 1037 mA h g^−1^ based on the weight of the CoFe_2_O_4_/GAs composites. The initial capacity loss can be probably associated with the formation of solid electrolyte interphase (SEI) layer on the surface of electrode in the first discharge step^47^. After 20 charge/discharge cycles, a high capacity of 830 mA h g^−1^ can still be retained. For comparsion, the mechanically mixed CoFe_2_O_4_/RGO composites were prepared (Figure S7). The mechanically mixed CoFe_2_O_4_/RGO composites demonstrate a relatively low capacity of 1772 mA h g^−1^, and the capacity decreases rapidly to 366 mA h g^−1^ after 20 charge/discharge cycles (Fig. 6b). In addition, the cycling performance of the CoFe_2_O_4_/GAs composites is greatly superior to that of the mechanically mixed CoFe_2_O_4_/RGO (Fig. 6c). The capacity of CoFe_2_O_4_/GAs is very stable at the current density of 0.1 A g^−1^ and the high reversible capacity of 830 mA h g^−1^ is still retained after 50 cycles, while the capacity of CoFe_2_O_4_/RGO rapidly decays from 1424 to 350 mA h g^−1^. The rate performances of CoFe_2_O_4_/GAs at the current rates of 0.1~2.0 A g^−1^ are depicted in Fig. 6d. Reversible capacity are retained at 602 mA h g^−1^ and 500 mA h g^−1^ at 0.5 A g^−1^ and 1.0 A g^−1^, respectively. Remarkably, a high reversible capacity of 340 mA h g^−1^ at a high rate of 2.0 A g^−1^ for the CoFe_2_O_4_/GAs composites can be delivered. Importantly, after charge/discharge tests at the high density current, the capacity of CoFe_2_O_4_/GAs can still return to the initial value, suggesting the high stability of CoFe_2_O_4_/GAs. As a comparison, the CoFe_2_O_4_/RGO composites demonstrated a much lower capacity of 15 mA h g^−1^ at a high rate of 2.0 A g^−1^ owing to the weak connections between CoFe_2_O_4_ nanoparticles and RGO sheets and the absence of 3D interconnected network. Figure 6e compares the Nyquist plots of electrodes of CoFe_2_O_4_/GAs and CoFe_2_O_4_/RGO. Apparently, the CoFe_2_O_4_/GAs electrode shows a much lower resistance than the CoFe_2_O_4_/RGO electrode (291 Vs. 538 Ω), which might be attributed to the excellent conductivity and electrochemical activity of CoFe_2_O_4_/GAs.

In order to further highlight advantage of CoFe_2_O_4_/GAs, we synthesized pure CoFe_2_O_4_ (Figure S3) and GAs (Figure S8) and tested their LIBs performance, respectively (Figure S9). The cycle stability of these three materials is given in Figure S9a. It can be observed that pure CoFe_2_O_4_ showed the low Li^+^ storage ability and bad stability due to the volume expansion and contraction associated with Li^+^ insertion/extraction during the charge/discharge processes. The GAs electrode gives an initial charge capacity of only 307 mA h g^−1^, much lower than that of CoFe_2_O_4_/GAs at the same current density and also lower than its theoretical value (372 mA h g^−1^). The rate capability of CoFe_2_O_4_/GAs, pure CoFe_2_O_4_ and GAs is compared in Figure S9b. Compared with pure CoFe_2_O_4_ and GAs, the CoFe_2_O_4_/GAs composites demonstrate a remarkably improved rate capability. The charge capacities of CoFe_2_O_4_/GAs at 0.1, 0.2, 0.5, 1.0, 2.0 A g^−1^ are 830, 710, 602, 500 and 340 mA h g^−1^, respectively, greatly higher than those of bare pure CoFe_2_O_4_ and GAs.

The outstanding electrochemical behavior of CoFe_2_O_4_/GAs with high capacity, stable cycle performance and excellent rate capacity, can be assigned to the following factors: (1) the unique 3D interconnected structure of CoFe_2_O_4_/GAs, which consists of macro- and mesopores on the graphene network, can effectively reduce the diffusion length for both electron and Li^+^ ions and provide multidimensional routes to facilitate the transport of electrons in the bulk electrode. (2) The large surface area of CoFe_2_O_4_/GAs can greatly improve ion adsorption for Li^+^ ions insertion/extraction during the charge/discharge process. (3) The strong coupling effect between CoFe_2_O_4_ and GAs can prevent large volume expansion/contraction and aggregation of CoFe_2_O_4_ nanoparticles associated with Li^+^ ions insertion/extraction during the discharge/charge process.

In conclusion, we have fabricated the CoFe_2_O_4_/GAs composites through a facile and cost-efficient hydrothermal self-assembly and freeze-drying two-step strategy. The generation of CoFe_2_O_4_ nanoparticles is accompanied with the reduction of GO under the hydrothermal condition and the obtained CoFe_2_O_4_ nanoparticles with diameters focused on around 9 nm are ultra-dispersed on the surface of RGO sheets. The CoFe_2_O_4_/GAs composites exhibit the superior Photo-Fenton activity for the degradation of MO in an aqueous system due to improved adsorption toward pollutants and high conversion efficiency of Fe^3+^/Fe^2+^. In addition, the magnetic recyclable usability of the CoFe_2_O_4_/GAs composites demonstrates over many successive reaction cycles. Besides of the promising application in Photo-Fenton reaction, the composites show excellent lithium storage performance with high reversible capacity and remarkable cyclic retention at each current density when used the anode material in LIBs. We believe that such multifunctional composites will have many potential practical applications in the environmental protection and energy development. It is also expected that the involved preparation method can be easily adapted and extended as a general approach to other systems for the preparation of highly dispersed nanoparticles on graphene aerogels.

## Method

### Materials

All chemicals, including Fe(NO_3_)_3_·9H_2_O (AR), Co(NO_3_)_2_·6H_2_O (AR), NaOH (AR), H_2_SO_4_ (AR), NaNO_3_ (AR), KMnO_4_ (AR), H_2_O_2_ (AR), acetonitrile (AR), hydrochloric acid (HCl) and ethanol (AR) were used as received without any further purification. Graphite powders were purchased from Sigma-Aldrich (St. Louis, MO), and ultrapure water was used for all experiments.

### Synthesis of Graphene Oxide (GO)

Graphene oxide (GO) was synthesized from natural graphite powder using a modified Hummers method^48^. Typically, 2 g graphite powders were added into a mixture of 50 mL H_2_SO_4_ and 1 g NaNO_3_. The solution was kept at 5 °C in an ice bath under vigorous stirring for 2 h. Thereafter, 6 g KMnO_4_ was added slowly into the mixture while the temperature was kept from exceeding 5 °C, then the temperature of the system was heated up to 35 °C and maintained for 2 h. Afterwards, 80 mL of water was slowly added and then the mixture was heated to 98 °C for 1 h. 280 mL of water and 80 mL of 30% H_2_O_2_ were added to end the reaction, followed by 5% HCl and filtration. Finally, the wet graphene oxide was freeze-dried at −60 °C for 24 h.

### Synthesis of the CoFe_2_O_4_/GAs composites

In a typical experiment, 75 mg GO powders were dispersed a mixed solvent containing 75 mL ethanol and 25 mL acetonitrile in an ultrasound bath for 90 min. Thereafter, 0.48 g Fe(NO_3_)_3_·9H_2_O and 0.173 g Co(NO_3_)_2_·6H_2_O were added into the solution under the stirring for 1 h, then 1 mL of NaOH (0.1 M) solution was added into the above solution while stirring. After stirring for 1 h, the suspension was centrifuged and washed with ethanol and water. The as-prepared product was re-dispersed in 25 mL of water followed by an ultrasonic treatment, which was then transferred into a 50 mL autoclave, and kept at 180 °C for 12 h. The aerogels was treated by freeze-drying to obtain a three-dimensional CoFe_2_O_4_/GAs composites. As a control experiment, two-dimensional (2D) CoFe_2_O_4_/reduced graphene oxide (RGO) composites were prepared by physically mixing CoFe_2_O_4_ and RGO. With the absence of GO, the pure CoFe_2_O_4_ nanoparticles were prepared by the similar method of preparation of CoFe_2_O_4_/GAs. Pure GAs were prepared by hydrothermal treatment of GO solution.

### Characterization

X-ray diffraction (XRD) patterns of all samples were collected in the range 10–80° (2θ) using a RigakuD/MAX 2550 diffract meter (Cu K radiation, λ = 1.5406 Å), operated at 40 kV and 100 mA. The morphologies were characterized by transmission electron microscopy (TEM, JEM2000EX). The particle size distribution curve was derived from 100 CoFe_2_O_4_ nanoparticles. The surface morphologies were observed by scanning electron microscopy (TESCAN nova Ш) and field emission scanning electron microscopy (FESEM, NOVA NanoSEM450). Raman measurements were performed at room temperature using Raman microscopes (Renishaw, UK) under the excitation wavelength of 532 nm. BET surface area measurements were carried out by N_2_ adsorption at 77 K using an ASAP2020 instrument. Thermogravimetric and differential thermal analyses were conducted on a Pyris Diamond TG/DTA (PerkinElmer) apparatus at a heating rate of 20 K min^−1^ from 40 to 800 °C in air flow.

### Photo-Fenton Reaction

The photocatalytic activity of each catalyst was evaluated by in terms of the degradation of methyl-orange (MO, 10 mg/L). The CoFe_2_O_4_/GAs powders were added into a 100 mL quartz reactor containing 75 mL MO solution. Prior to reaction, the initial pH value of the MO solution was adjusted to a certain pH value with 0.1 M HCl or 0.1 M NH_3_. Fenton reaction was initiated by adding a known concentration of H_2_O_2_ (a certain volume value, 30 wt %) to the solution. A 300 W Xe lamp (with AM 1.5 air mass filter) was used as a simulated solar light source. At the given time intervals, the analytical samples were taken from the mixture and immediately centrifuged before filtration through a 0.22 μm millipore filter to remove the photocatalysts. The filtrates were analyzed by recording variations in the absorption in UV-vis spectra of MO using a Cary 100 ultraviolet visible spectrometer. The leaching of Fe ions during reaction was analyzed using a Cary 100 ultraviolet visible spectrometer. In detail, a certain amount of solution was taken from the Photo-Fenton system. Next, a centrifuge separated the supernatant from the solution. And then, 1 mL 1, 10-phenanthroline monohydrate (0.5 wt%) as a testing Fe^2+^ reagent were added into 3 mL supernatant. After 15 minutes’ standing, the levels of ferrous iron were examined by using a Cary 100 ultraviolet visible spectrometer.

### Electrochemical Measurements

The electrochemical experiments were performed in coin-type cells. The working electrodes were prepared by mixing the hybrids, carbon black (Super-P), and poly-(vinyl difluoride) (PVDF) at a weight ratio of 80:10:10 to form slurry in N-methyl-2-pyrrolidinone (NMP), which was coated onto a copper foil (99.6%). Pure lithium foils were used as counter and reference electrodes. The electrolyte was consisted of a solution of LiPF_6_ (1 M) in ethylene carbonate (EC)/dimethyl carbonate (DMC) (1:1, in weight percent). The cells were assembled in an Ar-filled glove box with the concentrations of moisture and oxygen below 1 ppm. The electrochemical performance was tested on a LAND CT2001A battery test system in the voltage range of 0.01–3.00 V versus Li^+^/Li at room temperature.

## Additional Information

**How to cite this article**: Qiu, B. *et al*. Ultradispersed Cobalt Ferrite Nanoparticles Assembled in Graphene Aerogel for Continuous Photo-Fenton Reaction and Enhanced Lithium Storage Performance. *Sci. Rep.* **6**, 29099; doi: 10.1038/srep29099 (2016).

## Supplementary Material

Supplementary Information
