# Supplementary material for: Ultradispersed Cobalt Ferrite Nanoparticles Assembled in Graphene Aerogel for Continuous Photo-Fenton Reaction and Enhanced Lithium Storage Performance
Source: Sci Rep. 2016 Jul 4;6:29099. doi: 10.1038/srep29099 (PMC4931445; doi:10.1038/srep29099)
Supplement: Supplementary Information [file srep29099-s1.doc]

**Ultradispersed Cobalt Ferrite Nanoparticles Assembled in Graphene Aerogel for Continuous Photo-Fenton Reaction and Enhanced Lithium Storage Performance**

Bocheng Qiu, Yuanxin Deng, Mengmeng Du, Mingyang Xing* and Jinlong Zhang*.

Key Laboratory for Advanced Materials and Institute of Fine Chemicals, East China University of Science and Technology, 130 Meilong Road, Shanghai 200237, P.R. China


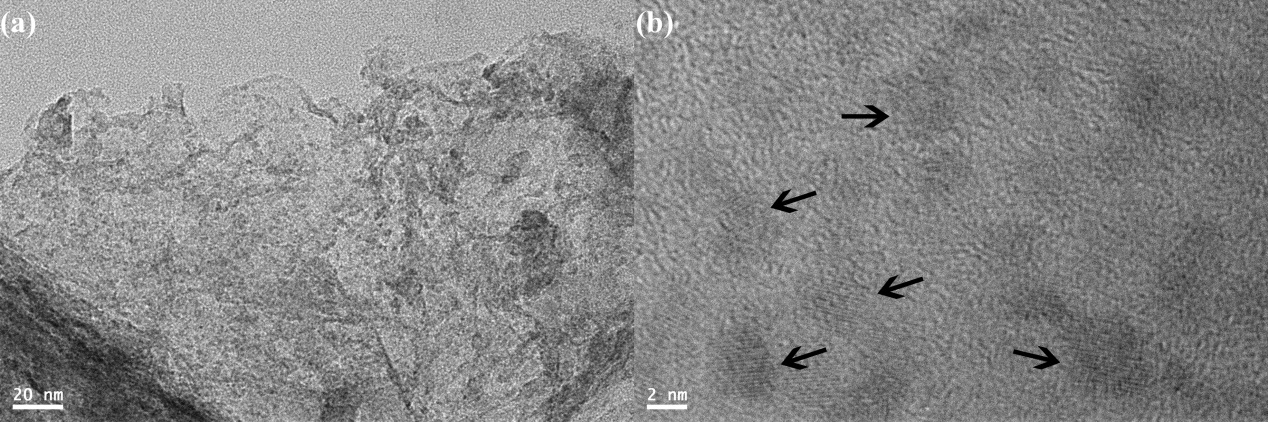


**Figure S1.** The HRTEM images of CoFe_2_O_4_ crystal seeds loaded on GO sheets (CoFe_2_O_4_/GO).


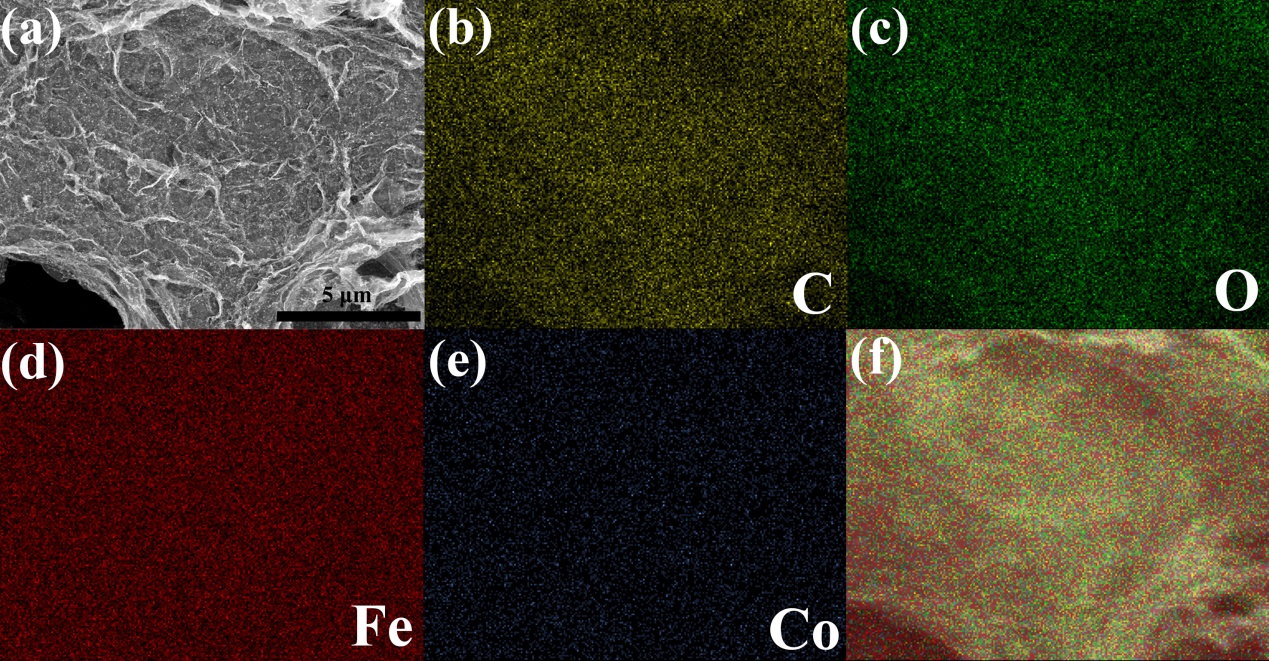


**Figure S2.** (a) The FESEM image, (b) C element, (c) O element, (d) Fe element, (e) Co element, (f) the overlapping of C, O, Fe, Co elements and FESEM mapping images of CoFe_2_O_4_/GAs.


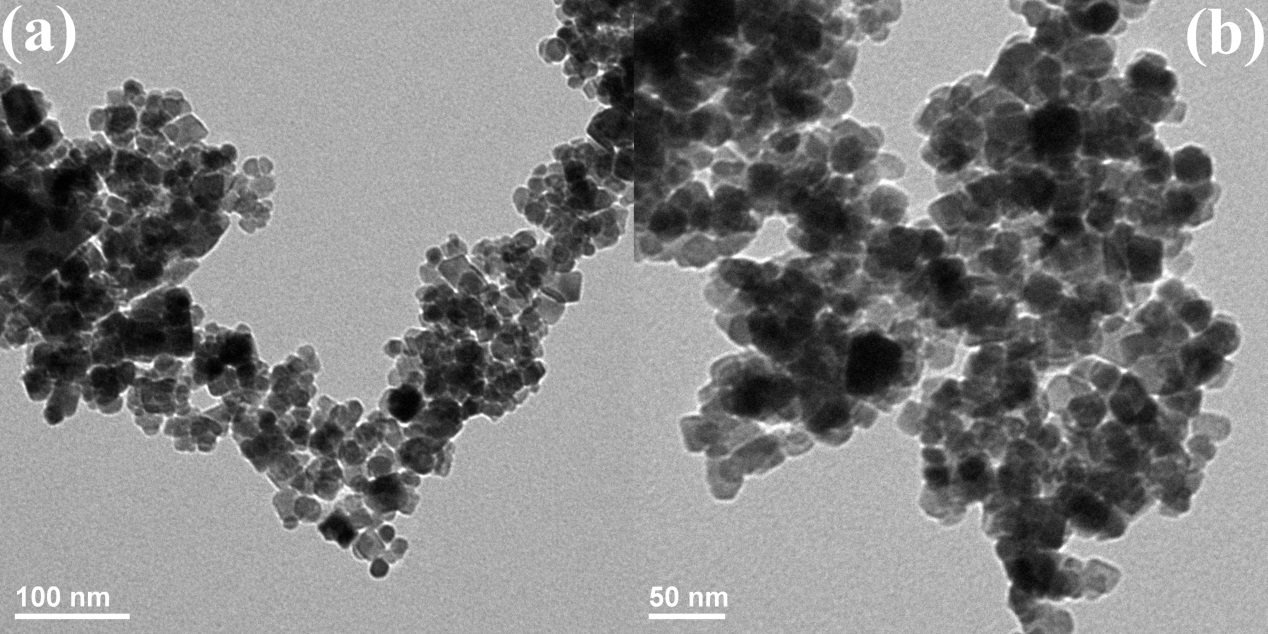


**Figure S3**. TEM images (a, b) of CoFe_2_O_4_


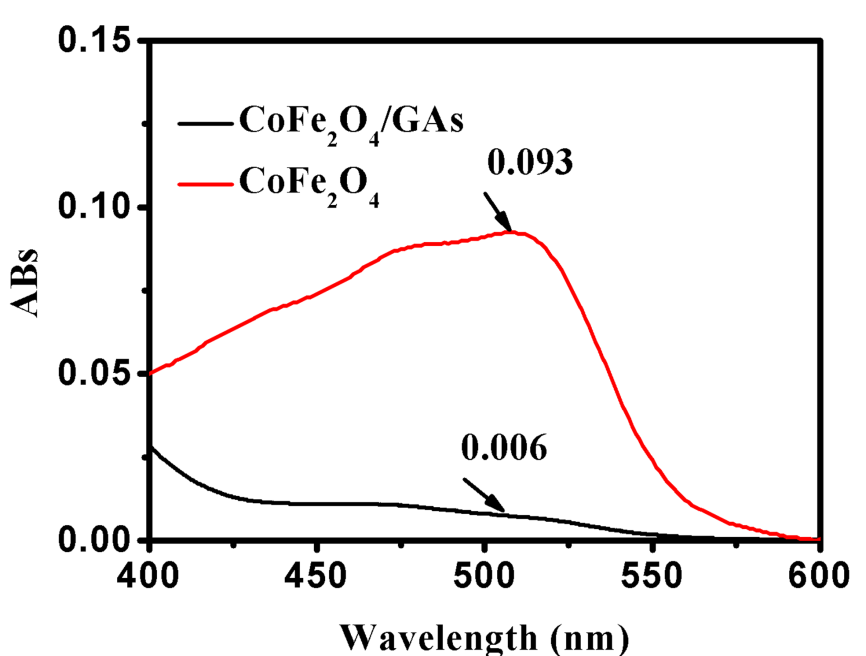


**Figure S4.** The detection of leaching of Fe^2+^ ions.


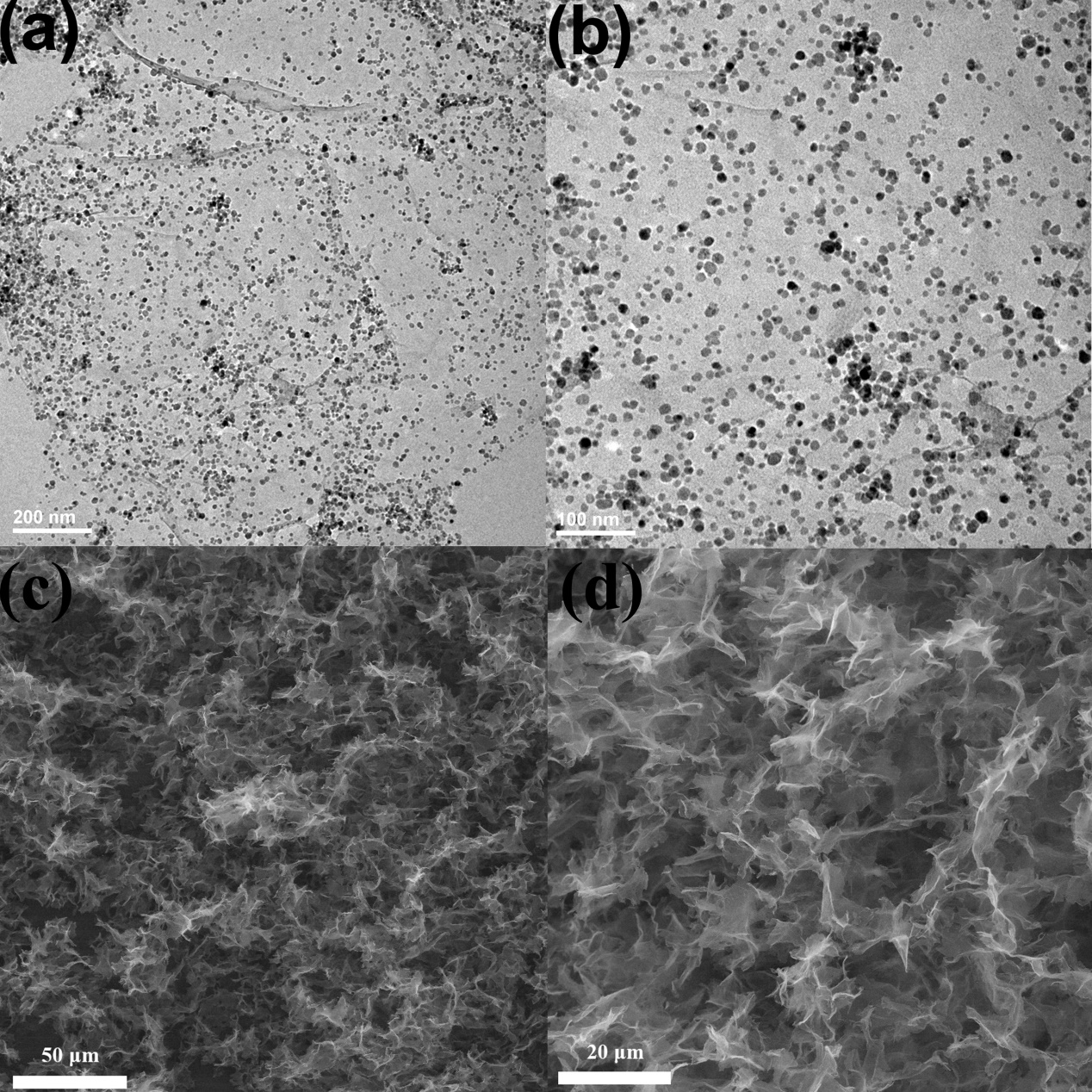


**Figure S5.** TEM (a, b) and SEM (c, d) images of CoFe_2_O_4_/GAs after 5 Photo-Fenton cycles


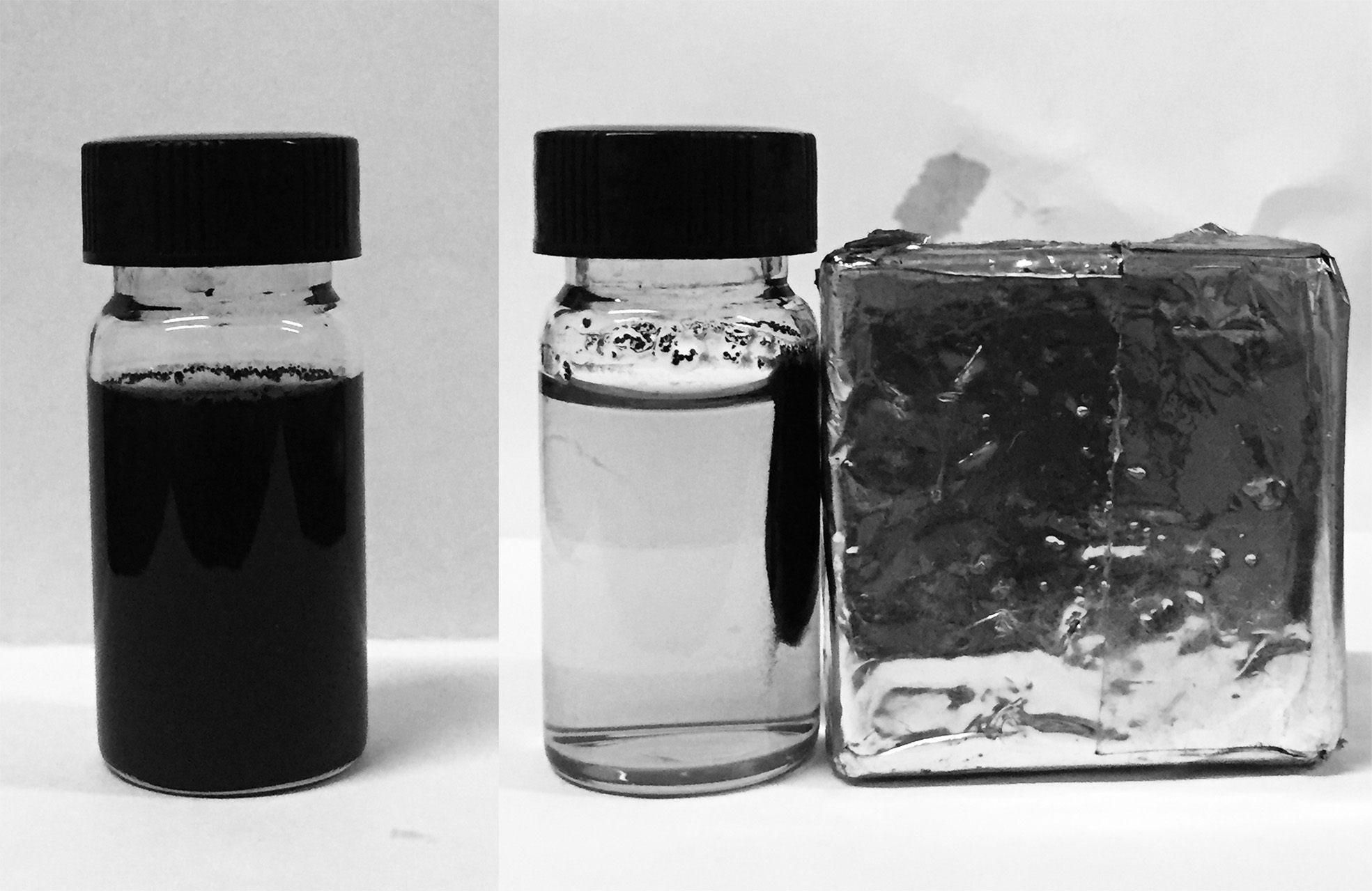


**Figure S6.** The magnetic separation property of CoFe_2_O_4_/GAs photocatalyst


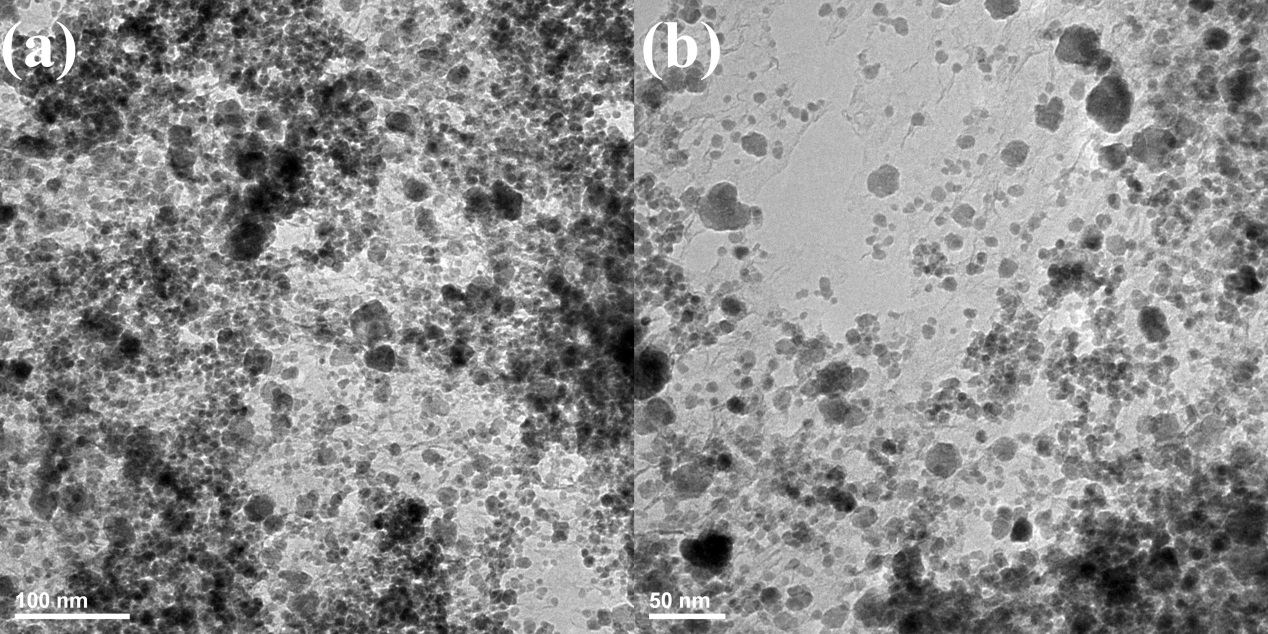


**Figure S7**. TEM images (a, b) of CoFe_2_O_4_/RGO


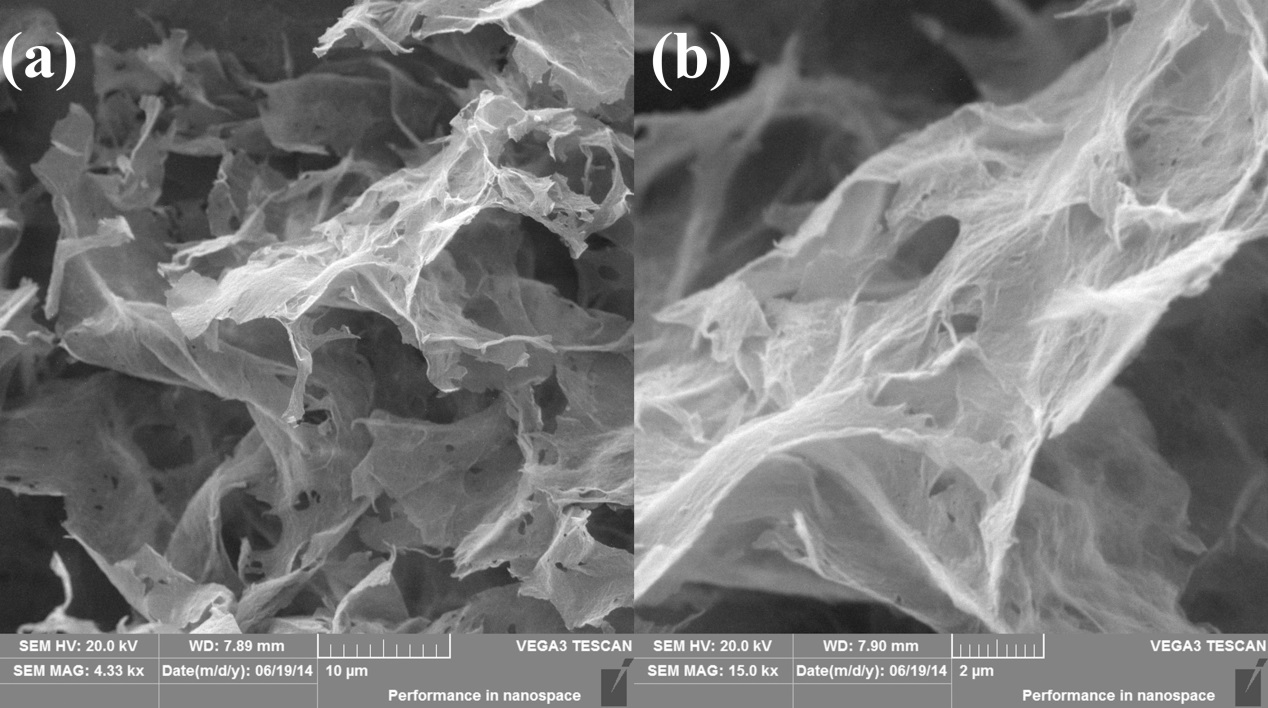


**Figure S8.** SEM images (a, b) of pure GAs.


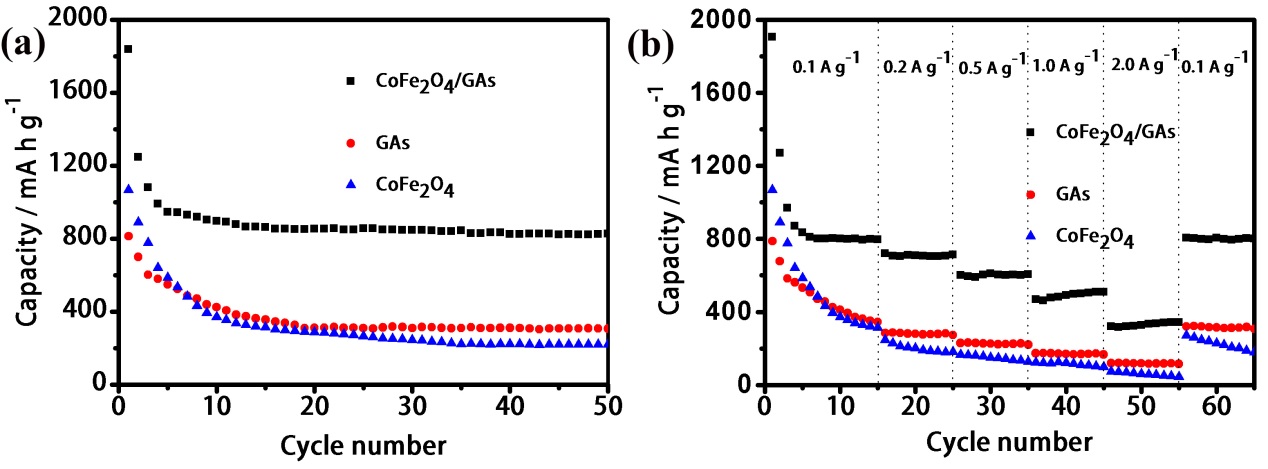


**Figure S9**. (a) Cycling performance of CoFe_2_O_4_/GAs composites, GAs and pure CoFe_2_O_4_ electrode at constant current densities of 0.1 A g^−1^. (b) Rate capability of CoFe_2_O_4_/GAs composites, pure GAs and pure CoFe_2_O_4_ electrode at each current density between 0.1 and 2.0 A g^-1^
